# The mediating role of HbA1c in the association between elevated low-density lipoprotein cholesterol levels and diabetic peripheral neuropathy in patients with type 2 diabetes mellitus

**DOI:** 10.1186/s12944-023-01865-5

**Published:** 2023-07-13

**Authors:** Hui Zhang, Yang Chen, Wenwen Zhu, Tong Niu, Bing Song, Hongxiao Wang, Wei Wang, Haoqiang Zhang

**Affiliations:** 1 Henan Key Laboratory of Rare Diseases, Endocrinology and Metabolism Center, The First Affiliated Hospital and College of Clinical Medicine of Henan of Science and Technology, Luoyang, China; 2 Department of Endocrinology, Anhui Provincial Hospital Affiliated to Anhui Medical University, Hefei, China; 3 Department of Endocrinology, The First Affiliated Hospital of USTC, Division of Life Sciences and Medicine, University of Science and Technology of China, Hefei, China; 4 Department of Endocrinology, Affiliated Zhongda Hospital of Southeast University, Nanjing, China; 5 Department of Endocrinology, The First Affiliated Hospital of Jinzhou Medical University, Jinzhou, China

**Keywords:** Low-density lipoprotein cholesterol, Diabetic peripheral neuropathy, Type 2 diabetes mellitus, Hyperglycemia, Nerve conduction velocity

## Abstract

**Background:**

Increased levels of low-density lipoprotein cholesterol (LDL-C) have been identified as one potential risk factor for diabetic peripheral neuropathy (DPN) in patients. The current study seeks to clarify the link between LDL-C, hyperglycemia, and DPN in patients with type 2 diabetes mellitus (T2DM).

**Methods:**

Here, a total of 120 T2DM individuals were recruited. These volunteers with T2DM were divided into 2 groups, based on the presence or absence of peripheral neuropathy. Additionally, their baseline characteristics were compared. Association among LDL-C and glycosylated hemoglobin (HbA1c) levels and DPN, particularly with respect to specific nerve conduction velocity were analyzed. To identify factors influencing DPN, regression was performed. Furthermore, mediation analysis was employed to evaluate the indirect, direct and total effects of LDL-C on specific nerve conduction velocity, with HbA1c serving as a mediator.

**Results:**

Compared to 55 patients without DPN, 65 patients with DPN demonstrated elevated levels of LDL-C and HbA1c. Both LDL-C and HbA1c have been found to be associated with reduced the motor fiber conduction velocities of Ulnar (or the Common peroneal) nerve in diabetic patients. HbA1c is one of the known risk factors for DPN in individuals with T2DM. Further mediation analysis revealed that the effect of LDL-C on the Ulnar (or the Common peroneal) nerve motor fiber conduction velocities are fully mediated by HbA1c in patients with T2DM.

**Conclusions:**

The impact of elevated LDL-C levels upon the Ulnar (or the Common peroneal) nerve motor fiber conduction velocities in patients with T2DM was found to be entirely mediated by increased HbA1c levels.

**Supplementary Information:**

The online version contains supplementary material available at 10.1186/s12944-023-01865-5.

## Background

T2DM is characterized by chronic hyperglycemia, insulin resistance, and commonly, lipid disorders [1]. Lipid abnormalities, particularly cholesterol metabolic disorders, have been identified as one of the factors influence complications of diabetes mellitus, including several kinds of neuropathy [2, 3]. Our previous study focusing on central nervous system diabetic complications demonstrated that hypercholesterolemia is one of the risk factors for cognition dysfunction in T2DM patients [4]. Moreover, our further exploration with 497 individuals showed that LDL-C levels have a U-shaped association with cognitive performance. Elevated LDL-C levels may impair executive function, while LDL-C deficiency could affect visual spatial function [5]. Besides central nervous system damage, diabetic patients exhibiting severe lipid metabolic disorders are at a higher risk of DPN than those without lipid disorders [6]. In addition, a study with cutaneous silent periods and autonomic tests demonstrated that mixed hyperlipidemia is associated with neuropathy in small fiber [7]. It is similar with another clinical research which indicated that individuals with normal weight had a significant lower prevalence of DPN (46.99%) than those (66.62%) with obesity [8]. More specific study showed that LDL-C is significantly correlated to the development of neuropathy with a OR = 1.22 (95% confidence interval of OR: 1.03–1.45) [9]. Another study with T2DM patients demonstrated that small LDL-C particle size is associated with neuropathy independently [10]. However, a recent study indicated that decreased level of serum cholesterol is related to declining nerve fibers conduction velocities and amplitudes as well as increased amount of nerve lesions in patients with T2DM [11].

Based on the aforementioned description, patients diagnosed with T2DM exhibit a key characteristic of chronic hyperglycemia. Notably, the regulation of plasma glucose levels has been linked with DPN. It has been suggested that glycemic variability is intimately linked with DPN in T2DM patients [12]. Additionally, prior investigations have demonstrated that elevated levels of HbA1c (associated with poorly controlled plasma glucose) are related to a markedly elevated risk of DPN in diabetic patients [13]. Furthermore, lipid profiles may serve as a potential predictor of the plasma glucose control status in individuals with T2DM [14].

The impact of cholesterol levels in the peripheral blood on DPN in patients with T2DM is still uncertain. This present work aims to clarify the link between LDL-C levels and peripheral neuropathy, specifically focusing on the damage to certain nerve fibers. Although both LDL-C and HbA1c have been associated with DPN and identified as risk factors in diabetic patients, lipid profiles are also related to the regulation of plasma glucose. Nevertheless, the link among LDL-C, HbA1c, and DPN requires further investigation. Therefore, this present research aims to analyze the impact of elevated LDL-C upon DPN in individuals with T2DM, taking into account the effect of HbA1c as a potential mediator through medication analysis.

## Methods

### Experiment design

This study was conducted at The First Affiliated Hospital of University of USTC. An amount of 120 patients were recruited for this present study. All these individuals were certified the standard criteria for T2DM. Of the recruited individuals, 65 were diagnosed with DPN and 55 were found to be without DPN.

### Ethics

Prior to the commencement of the experiment, all participants were informed of the clinical research process. Informed consent was obtained from each volunteer, as evidenced by their signature. Additionally, the Ethics the Committee for Medical Research of our institution approved this present work (Approval No.: 2023-RE-013). This cross-sectional study adhered to the principles in Declaration of Helsinki.

### Inclusion and exclusion criteria

All the participants in this study were diagnosed with T2DM and met the criteria set forth by the World Health Organization [15]. Patients with T2DM were included in this study. The exclusion criteria were similar with our previous study [16] and defined specially described as fellows: (a) other types of diabetes (including type 1 diabetes mellitus, gestational diabetes, and specific types of diabetes); (b) diabetes with acute complications; (c) neuropathy caused by other diseases or drugs; (d) severe vascular disease (e.g. venous embolism, lymphangitis); (e) neurotoxicity caused by drugs; (f) other undefined disease or drugs may influence neuropathy; f) any amputation; (g) diagnosed thyroid disease (except for thyroid nodules); g) smoking and alcohol abuse; (h) any other undefined condition influence the performance of neurophysiological examinations. The Toronto Consensus criteria was used to diagnosis the presence of DPN [17]. The T2DM patients diagnosed with DPN were divided into the DPN group, while those who did not meet the diagnostic criteria for DPN were designated as the control group.

### Clinical data

The present study collected data on age, gender, height, weight, and duration of diabetes mellitus (DM). Information on drug administration, particularly statins and other lipid-lowering drugs, was also recorded. Weight (kg)/height (m)^2^ was used to calculate the body mass index (BMI). Dry chemical method was used to measure the levels of Fasting plasma glucose (FPG). Microcolumn ion-exchange chromatography was employed to detect HbA1c levels. High-density lipoprotein cholesterol (HDL-C), LDL-C, total cholesterol (TC), triglyceride (TG) and fasting C peptide (FCP) were also observed from blood samples. Insulin resistance was calculated according to the HOMA-IR by 1.5 + FBG (mmol/L) × FCP (pmol/L)/2800 [18]. These measurements were conducted in The First Affiliated Hospital of USTC, Center Laboratory for medical usage. These data were collected for further analysis.

### Neurophysiological tests

Neurophysiological examinations were conducted on patients diagnosed with T2DM at our hospital. These examinations were carried out in the Electrophysiology room by trained personnel, using an electromyographic evoked potential meter in accordance with the manufacturer’s protocol (Natus Neurology, USA). The data of nerve conduction velocity was recorded from the medical histories of the enrolled T2DM patients. The average values of the bilateral nerve conduction velocities were computed for further analysis.

### Statistical methods

IBM SPSS Statistics version 26.0 were used to analysis the data in this study. Variables with a normal distribution, including FPG, LDL-C, Tibial nerve motor conduction velocity, as well as sensory fibers conduction velocities of the Ulnar, Radial, Median nerve, and Sural nerve, were described as mean ± standard deviation. The differences of these variables between diabetic patients with and without DPN were compared by Student’s t-tests. Age, height, weight, BMI, duration of DM, and HbA1c, as well as motor conduction velocities of the Ulnar nerve, Radial nerve, Median nerve, and Common peroneal nerve, were described as median and interquartile range, and their differences between diabetic patients with and without DPN were compared using nonparametric Mann-Whitney U tests, as these variables were symmetrically distributed. Gender and information about the use of lipid-lowering drugs were expressed as a percentage, and their differences between T2DM patients with and without DPN were compared using the Chi-squared test because they were binary variables. To investigate the relationships between DPN and LDL-C (or HbA1c), we conducted Pearson correlation and Binary logistic regression analyses. We employed mediation analysis to assess the total, direct, and indirect effects of LDL-C on specific nerve conduction velocity, with HbA1c serving as a mediator. In this approach, we decomposed the “total effect” into a “direct effect” (not mediated by HbA1c) and an “indirect effect” (mediated by HbA1c), and we calculated the mediation effect as indirect effect/total effect × 100%. The significance of the mediation effect was evaluated using the bootstrap test. We conducted analysis to explore whether HbA1c mediated the impact of LDL-C upon DPN. *P* < 0.05 was defined as statistical significance.

## Results

### Clinical data of T2DM patients

The clinical data of participants with and without DPN in the study were summarized in Table 1. As anticipated, age and gender did not differ significantly between individuals with or without DPN (all *P* > 0.05). Additionally, there were no statistical differences in the clinical data, including height, weight, BMI, and duration of DM, between the two groups (all *P* > 0.05). Furthermore, the biochemical data presented in Table 1 revealed that the levels of FPG and HbA1c were lower in participants in control group than those with DPN (all *P* < 0.05). Although there was no statistical significance in the levels of LDL-C between the two groups, the level of LDL-C was lower in patients without DPN than it in the DPN group (*P* = 0.052). Nevertheless, there was no statistical difference in lipids levels (including TG, TC, and HDL-C) and HOMA-IR between the two groups (all *P* > 0.05). Given that cholesterol metabolism was a focus of this research, the lipid-lowering drugs usage was also recorded. However, no differences in the use of statins or other lipid-lowering drugs were observed between diabetic individuals with or without peripheral neuropathy (all *P* > 0.05).

**Table 1:** Comparation of clinical parameters and neurophysiological test results between Control and DPN group

	Control group (n = 55)	DPN group (n = 65)	*P*
Age (years)	58 (51–64)	57 (51.5–67)	0.666^b^
Female (n, %)	20, 36.36	24, 36.92	0.949c
High (cm)	169 (160–172)	167 (160.5-173.5)	0.870
Weight (kg)	70 (60-77.5)	69 (60.25–81.5)	0.802
BMI (kg /m^2^)	24.62 (22.66–26.54)	24.88 (21.91–27.84)	0.796^b^
Duration of DM (years)	8 (3–12)	10 (1-18.5)	0.346^b^
Duration of Hypertension (years)	0.00 (0.00–10.00)	0.00 (0.00-9.50)	0.963^b^
FPG (mmol/l)	8.92 ± 3.08	10.21 ± 4.02	0.054^a^
FCP (nmol/l)	0.40 (0.24–0.55)	0.27 (0.17–0.47)	0.058^b^
HOMA-IR	2.62 (2.17–3.35)	2.41 (2.12–3.27)	0.464^b^
HbA1c (%)	9.0 (7.3–10)	10.3 (8.85–12.2)	0.001^b*^
TG (mmol/l)	1.77 (1.25–2.45)	1.67 (1.10–2.54)	0.431^b^
TC (mmol/l)	4.51 (3.7–5.02)	4.62 (4.01–5.35)	0.111^b^
HDL-C (mmol/l)	0.85 (0.68–1.06)	0.87 (0.69–1.13)	0.484^b^
LDL-C (mmol/l)	2.34 ± 0.87	2.68 ± 0.98	0.052^a^
Statin (n, %)	25, 45.45	32, 49.23	0.680^c^
Other lipid-lowering drugs (n, %)	4, 7.27	6, 9.23	0.699^c^
**Motor conduction**			
Ulnar nerve (m/s)	61.20 (59–63)	54.40 (51.35–59.80)	0.000^b*^
Radial nerve (m/s)	64.60 (62.55–66.20)	62.50 (60.85–64.62)	0.001^b*^
Median nerve (m/s)	60.00 (55.65–62.15)	53.50 (49.75–56.03)	0.000^b*^
Tibial nerve (m/s)	47.28 ± 3.17	41.00 ± 3.63	0.000^a*^
Common peroneal nerve (m/s)	46.75 (44.85–48.50)	42.15 (38.88–44.55)	0.000^b*^
**Sensory conduction**			
Ulnar nerve (m/s)	55.94 ± 4.79	48.72 ± 5.90	0.000^a*^
Radial nerve (m/s)	57.17 ± 8.27	50.71 ± 6.67	0.000^a*^
Median nerve (m/s)	56.83 ± 5.61	45.47 ± 7.60	0.000^a*^
Sural nerve (m/s)	52.05 ± 4.96	44.68 ± 5.39	0.000^a*^

The data are presented as n (%), or the median (inter-quartile range) unless otherwise specified

a Student’s t test was employed for normally distributed variables

b The Mann-Whitney U test was employed for asymmetrically distributed variables

c The Chi-square test was employed for categorical variables

^*^P < 0.05

Abbreviations: DPN, diabetic peripheral neuropathy; BMI, body mass index; DM, diabetes mellitus; FPG, fasting plasma glucose; HbA1c, glycosylated hemoglobin; FCP, fasting C-peptide; TG, triglycerides; TC, total cholesterol; LDL-C, low density lipoprotein cholesterol; HDL-C, high density lipoprotein cholesterol

### Difference of neurophysiological examination results in two groups

In order to validate DPN observed in diabetic patients, a neurophysiological assessment was conducted. Intriguingly, all motor fibers, encompassing the Ulnar, Radial, Median, and Tibial nerve, as well as sensory fibers, including the Ulnar, Radia, Median, and Sural nerve, exhibited reduced conduction velocities in T2DM patients suffering from DPN in comparison to those without DPN (as presented in Table 1) (all *P* < 0.05).

### Pearson correlation between LDL-C and nerve conduction velocity

**Fig. 1:**
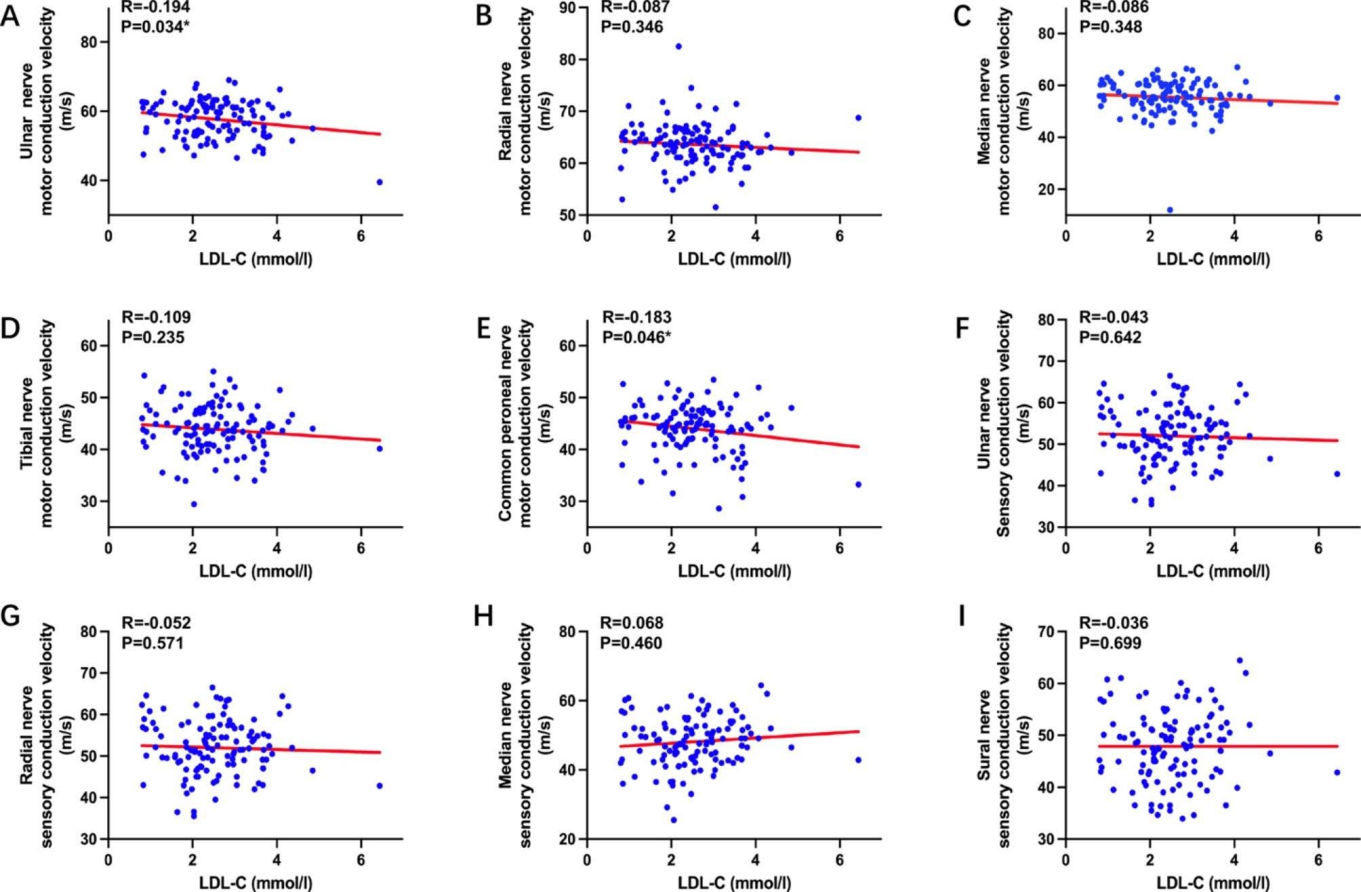
Pearson correlation between LDL-C and nerve conduction velocity in patients with T2DM ^*^P < 0.05 Abbreviations: LDL-C, low density lipoprotein cholesterol; T2DM, type 2 diabetes mellitus

In order to clarify the link between LDL-C and DPN, Pearson correlation analysis was employed. The results of this analysis found that LDL-C levels are negatively associated with both the Ulnar (*P* = 0.034) and Common peroneal (*P* = 0.046) nerve motor fiber conduction velocities, as depicted in Fig. 1.

### Pearson correlation between HbA1c and nerve conduction velocity

**Fig. 2:**
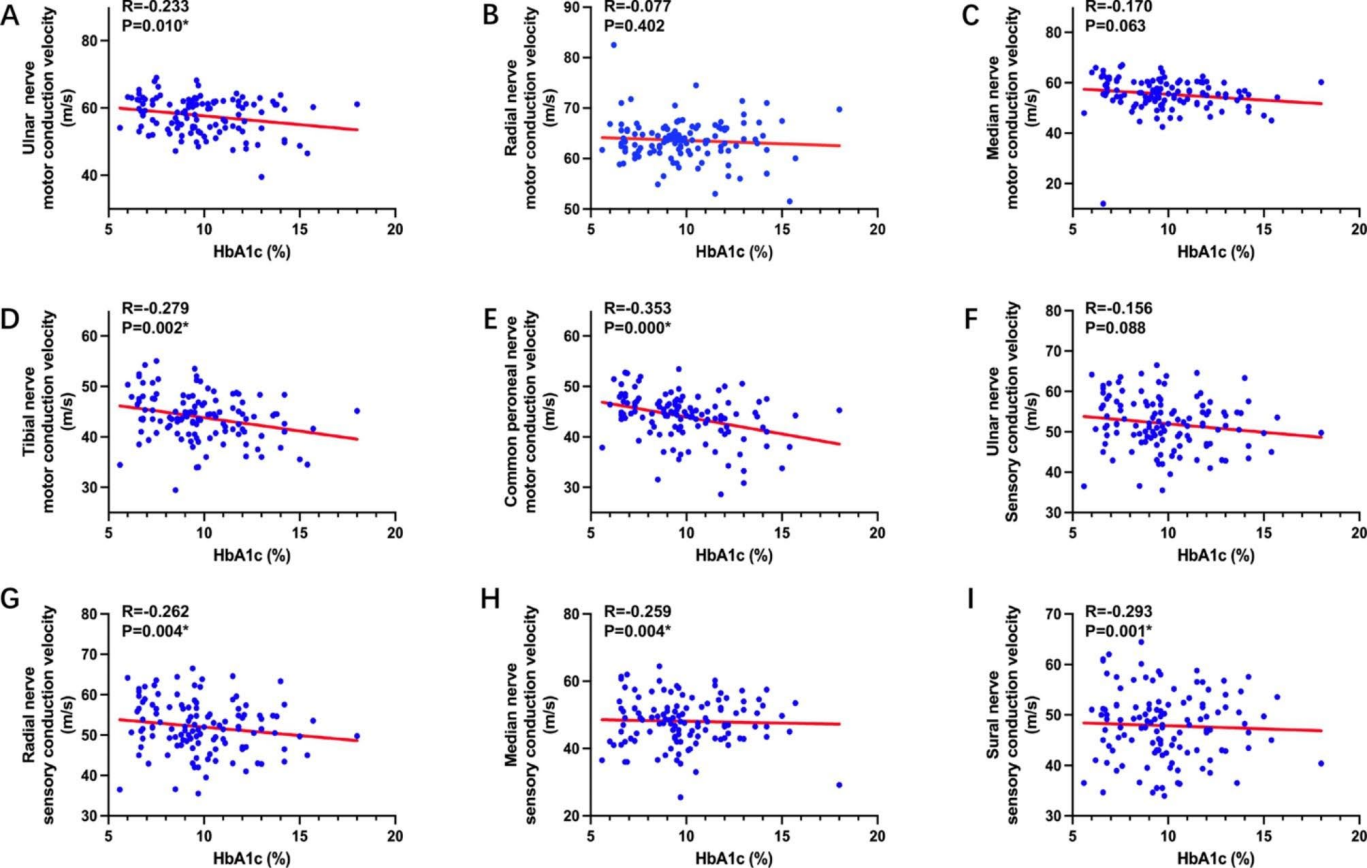
Pearson correlation between HbA1c and nerve conduction velocity in patients with T2DM ^*^P < 0.05 Abbreviations: HbA1c, glycosylated hemoglobin; T2DM, type 2 diabetes mellitus

In order to explore the link between HbA1c and DPN, a Pearson correlation analysis was conducted. Remarkably, the results revealed that HbA1c levels are also negatively correlated to motor fiber conduction velocities of the Ulnar, Tibial, and Common peroneal nerve (all *P* < 0.05). Furthermore, levels of HbA1c were identified to be negatively associated with sensory fiber conduction velocities of the Radial, Median, and Sural nerve (all *P* < 0.05) (demonstrated in Fig. 2).

### Binary logistic regression for risk factors of DPN

To explore the risk factors of DPN in individuals diagnosed as T2DM, we conducted a binary logistic regression analysis. The results indicated that HbA1c was one risk factor for DPN in volunteers diagnosed as T2DM (OR = 1.318, *P* = 0.002), while LDL-C was not a risk factor for DPN after adjusting for HbA1c levels (OR = 1.491, *P* = 0.056), as shown in Table 2. These findings suggest that glycemic control, as measured by HbA1c, may involve in DPN in T2DM individuals.

**Table 2:** Binary logistic regression analysis for risk factors of DPN in T2DM patients

	*P*	OR	95% confidence interval of OR	
			Lower	Higher
HbA1c	0.002^*^	1.318	1.109	1.567
LDL-C	0.056	1.491	0.990	2.244

^*^P < 0.05

Abbreviations: DPN, diabetic peripheral neuropathy; T2DM, type 2 diabetes mellitus; HbA1c, glycosylated hemoglobin; LDL-C, low density lipoprotein cholesterol

### Regression analysis for the medicating role of HbA1c in the association between LDL-C and DPN

**Fig. 3:**
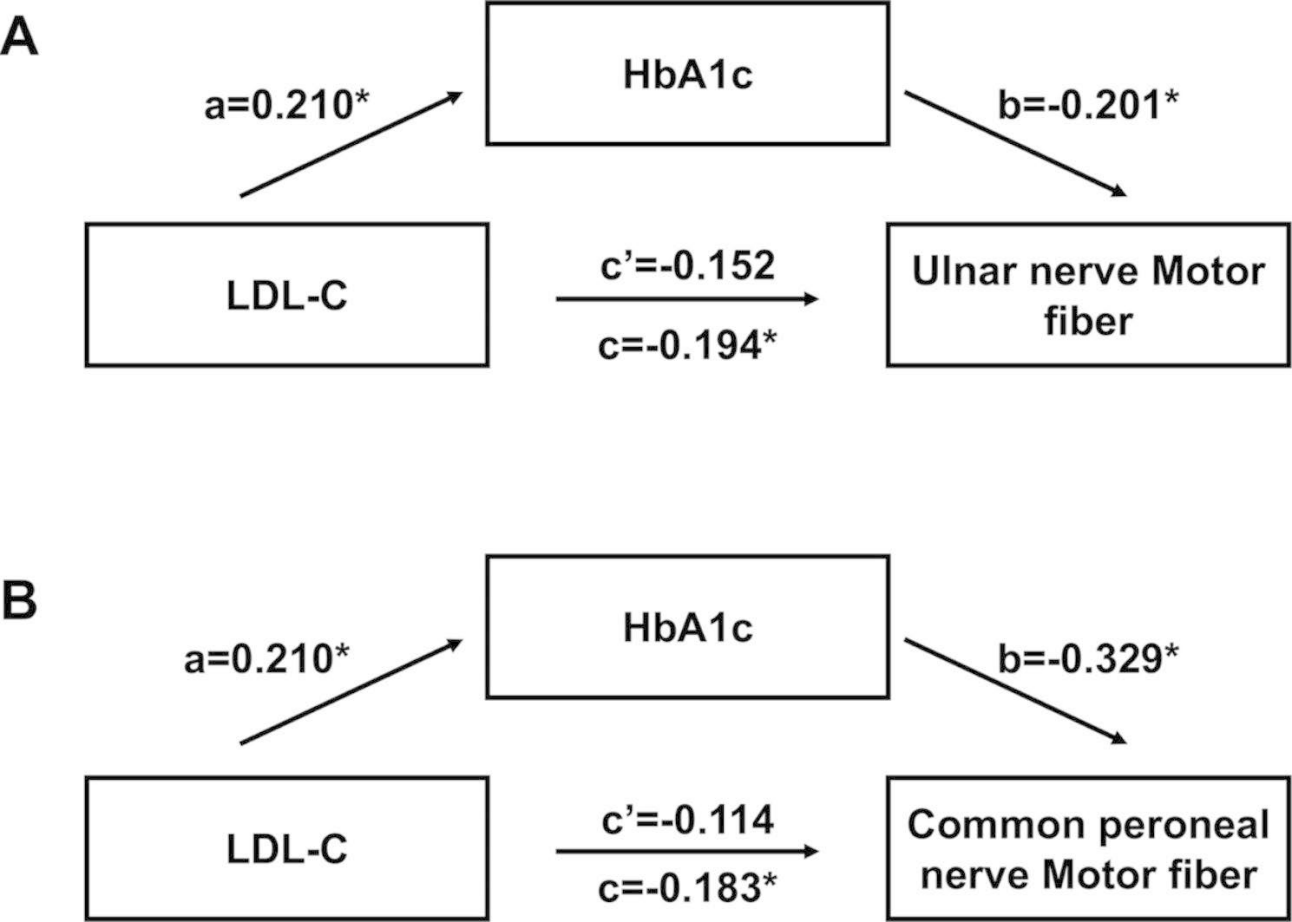
Mediating role of HbA1c in the relationship between LDL-C and DPN ^*^P < 0.05 Abbreviations: LDL-C, low density lipoprotein cholesterol; HbA1c, glycosylated hemoglobin; DPN, diabetic peripheral neuropathy

As previously mentioned, there exists an association not only between motor fiber conduction velocities of the Ulnar (or Common peroneal) nerve and LDL-C, but also a correlation between them and HbA1c. To investigate the mediating role of HbA1c in the impact of LDL-C upon DPN, we conducted a three-step regression analysis. Firstly, we found that LDL-C was not only a factor influencing the motor fiber conduction velocity of the Ulnar nerve, but also the Common peroneal nerve (*P* = 0.034 and 0.046, respectively) (Table 3). Secondly, we identified that LDL-C was a risk factor influencing the HbA1c levels of patients with T2DM (*P* = 0.022) (Table 4). Thirdly, multiple linear regression demonstrated that when HbA1c was entered as an independent variable, LDL-C was neither a factor influencing the motor fiber conduction velocity of the Ulnar nerve, nor Common peroneal nerve (*P* = 0.098 and 0.197, respectively) (Table 5). Furthermore, our mediation analysis (Fig. 3) indicated that in participants, the association between LDL-C and the Ulnar nerve motor fiber conduction velocity was fully mediated by HbA1c (Fig. 3A). Additionally, we found that HbA1c played a mediating role in the association between elevated LDL-C levels and decreased Common peroneal nerve motor fiber conduction velocity in individuals with T2DM (Fig. 3B).

**Table 3:** Linear regression analysis for factors influencing the nerve conduction velocity of T2DM patients

	*P*	β	95% confidence interval of β	
			Lower	Higher
Ulnar nerve motor				
LDL-C	0.034^*^	-1.109	-2.133	0.086
Common peroneal nerve motor				
LDL-C	0.046^*^	-0.891	-1.764	0.018

^*^P < 0.05

Abbreviations: LDL-C, low density lipoprotein cholesterol; T2DM, type 2 diabetes mellitus

**Table 4:** Linear regression analysis for factor influencing the HbA1c level of T2DM patients

	*P*	β	95% confidence interval of β	
			Lower	Higher
LDL-C	0.022^*^	0.539	0.081	0.996

^*^P < 0.05

Abbreviations: HbA1c, glycosylated hemoglobin; T2DM, type 2 diabetes mellitus

**Table 5:** Multiple linear regression analysis for factors influencing the nerve conduction velocity of T2DM patients

	*P*	β	95% confidence interval of β	
			Lower	Higher
Ulnar nerve motor				
LDL-C	0.098	-0.868	-1.897	0.162
HbA1c	0.029^*^	-0.449	-0.850	-0.048
Common peroneal nerve motor				
LDL-C	0.197	-0.555	-1.403	-0.294
HbA1c	0.000^*^	-0.624	-0.954	-0.294

^*^P < 0.05

Abbreviations: HbA1c, glycosylated hemoglobin; T2DM, type 2 diabetes mellitus

## Discussion

According to estimates, there are 451 million adults worldwide who have been diagnosed with diabetes. It is projected that by 2045, this number will increase to 693 million [19]. In China alone, the estimated prevalence of diabetes among adults is 10.9%, equating to a total of approximately 1.09 billion individuals with the disease in mainland China [20]. Previous study demonstrated that insulin resistance [21, 22], oxidative stress [23, 24], and demyelination [25] were regarded as important roles on the pathogenesis of DPN. Specifically, insulin resistance is identified as a potential risk factor for diabetic neuropathy. Our previous studies have found a correlation between insulin resistance and cognitive impairment in individuals with diabetes. Diabetic cognitive impairment represents a complication of the central nervous system in diabetes patients [26]. However, in this study, although we took insulin resistance into account in this work, due to many of our diabetes patients receiving insulin therapy, we evaluated insulin resistance based on FCP levels and FPG levels, rather than fasting insulin levels and FPG levels. We did not find any difference in insulin resistance between the control group and the DPN group. Based on the information above, although we did not find significant statistical significance, we do not deny the role of insulin resistance in DPN. Additionally, our recent research findings, published recently, have established an association between lower levels of uric acid and peripheral neuropathy in diabetes. Uric acid serves as a natural antioxidant, protecting the body against oxidative stress [27].

Hyperglycemia is one of the most important factors related to diabetic complication [28, 29]. Diabetes duration is known to be one risk factor for DPN in T2DM individuals [30]. In this research, we observed increased levels of HbA1c in T2DM participants with peripheral neuropathy. Additionally, we demonstrated that elevated levels of HbA1c are correlated with decreased conduction velocity in the motor fibers of the Ulnar, Tibial, and Common peroneal nerve, as well as in the sensory fibers of the Radial, Median, and Sural nerve. These findings are agreed with a recent study, which indicated that elevated HbA1c levels are linked to DPN in patients with diabetes [31].

Despite observing elevated LDL-C levels in patients with DPN, compared to those in the control group, there is no statistical significance. Similarly, slightly increased LDL-C levels were found in diabetic patients with and without cognitive dysfunction, another neuronal complication in the central nervous system [5]. However, no significant difference in LDL-C levels was found in overall patients with cognitive impairment. Elevated LDL-C level may influence cognitive function in diabetic patients with extremely high levels of LDL-C. Some in vitro and in vivo studies have identified mechanisms linking lipid metabolism to DPN [2]. Cholesterol control seems to have a beneficial effect on DPN in patients with diabetes [32], although these studies have mainly focused on the effect of HDL-C on DPN. There is limited research on the link between levels of LDL-C and the occurrence of DPN. In this study, we observed that increased LDL-C level is one risk factor for DPN in individuals with T2DM. Furthermore, association analysis revealed that LDL-C is associated with the Ulnar and Tibial nerve motor fiber conduction velocities.

In the present study, aside from the correlation between LDL-C and DPN, the difference in levels of LDL-C were found to be similar to the levels of HbA1c in both groups. Further association analysis confirmed the association between levels of HbA1c and LDL-C in all diabetic individuals and those with peripheral neuropathy (Supplementary Table 1). Notably, disorders in lipoprotein metabolism are common among individuals with T2DM [33], and studies have linked this to the formation of advanced glycation end products (AGEs) in hyperglycemic conditions. Chang et al. demonstrated that diabetic patients exhibit a worse lipid profile that is associated with increased AGEs [34], which is formed in the condition of hyperglycemia. Basic experiments further revealed that AGEs activate the signaling pathway of sterol regulatory element-binding protein that is involved in cholesterol metabolism [35–37].

The aforementioned information illustrates the existence of a correlation between the motor fiber conduction velocity in the Ulnar or Common peroneal nerve and LDL-C levels in T2DM patients. Moreover, a correlation was also observed between the conduction velocities of motor fiber in the Ulnar or Common peroneal nerve and the levels of HbA1c in T2DM patients. Notably, LDL-C levels were identified to be linked with HbA1c levels in T2DM individuals. Therefore, a three-step regression was employed to clarify the mediating role of HbA1c in the relationship between elevated LDL-C levels and DPN in T2DM participants. Intriguingly, the impact of elevated LDL-C levels upon the conduction velocity of motor fibers in the Ulnar or Common peroneal nerves was completely mediated by HbA1c in individuals with T2DM. From a statistical perspective, we have indeed found that the impact of LDL-C on DPN is mediated by HbA1c, and this mediation effect is considered fully. This is solely a statistical result. Our main conclusion aims to emphasize the significant role of HbA1c in this process. However, we cannot disregard the possibility of LDL-C influencing DPN through other pathways.

### Study strengths and limitations

The present study described the characteristics of T2DM individuals with and without peripheral neuropathy, specifically focusing on nerve conduction velocities and levels of LDL-C and HbA1c. Furthermore, the study examines the relationship between LDL-C and HbA1c with nerve conduction velocities as well as the mediating role of HbA1c in the effect of elevated LDL-C levels on nerve conduction velocities in T2DM patients. However, some limitations need to be addressed. Firstly, this is a small sample cross-sectional study, and thus, only an association, rather than a causal relationship, between LDL-C (or HbA1c) and nerve conduction velocities can be determined. Secondly, it is imperative to acknowledge the significant impact of Oxidized low-density lipoprotein cholesterol (oxLDL-C) on diabetic complications [38]. However, our study is a retrospective cross-sectional investigation, and our data are derived solely from patients’ clinical records. The detection of oxLDL-C is not routinely performed in clinical practice. This is a limitation of our research. Furthermore, the non-detection of oxLDL-C can be attributed to the fact that our study aims to investigate the impact of LDL-C levels on DPN. LDL-C is a widely recognized clinical index, and understanding its influence on DPN holds greater clinical significance. Due to previous research findings demonstrating the significant impact of smoking on diabetic neuropathy [39], we have excluded smokers from our experimental design. Similarly, patients with alcohol abuse have also been excluded from the study for that there may be a link between drinking and DPN [40, 41]. Nonetheless, it would be highly meaningful to specifically analyze the effects of smoking and alcohol consumption on DPN by considering these factors. However, considering smoking and alcohol consumption (including duration and amount) is a complex task, and it is not the primary focus of our study. Additionally, we have not considered patients with indirect exposure to tobacco. This is due to research indicating that second-hand smoking may have an influence on the patients’ nervous system [42]. Waist circumference and waist-to-hip ratio were not addressed in our study because this information was not available in our patients’ medical records. Although we did consider BMI, there were no significant differences in BMI between the two groups of patients. Similarly, high-sensitivity C-reactive protein is not a routine test in our clinical practice, so there is no information on high-sensitivity C-reactive protein in our medical records. Therefore, we described these considerations as limitations of this work. Likewise, the impact of blood pressure on DPN should not be disregarded [43]. Here, we have compared the duration of hypertension in two patient groups. However, no significant differences were observed between the two groups. For many patients did not suffer from hypertension, we did not extensively record detailed blood pressure data during the time they were hospitalized. Due to the fluctuating nature of blood pressure, a targeted experimental design is necessary for a comprehensive investigation of its influence. Furthermore, due to our divergent research objectives, there exist numerous factors that were not addressed in our study, which may have an impact on diabetic neuropathy. These factors encompass hyponatremia [44], sarcopenia and muscle mass [45], abnormal albuminuria and glomerular filtration rate [46], as well as the Urine Albumin-to-Creatinine Ratio [47]. Thirdly, as an important factor influencing cholesterol metabolism, lipid-lowering drugs were considered. However, information regarding their form and dosage was not collected. Common neurophysiological examinations encompass two types: electromyography and somatosensory evoked potentials. Electromyography is widely utilized, while somatosensory evoked potentials can reflect damage to small nerves. Concerning electromyography, the examination results consist of three aspects: latency, amplitude, and nerve conduction velocity. Analyzing the results in relation to other neurophysiological parameters may yield more intriguing findings. However, in this study, our primary focus lies on nerve conduction velocity as the main analytical indicator. This limitation should also be acknowledged in our research. Lastly, this study solely focuses on the effect of increased LDL-C levels on DPN. So, the deficiency of cholesterol was not taken into account in this present research.

### Comparisons with other studies and what does the current work add to the existing knowledge

In general, previous studies mostly clarified the risk factors for DPN in diabetic individuals. This work not only investigated the risk factors for DPN, but also determined the association between LDL-C and specific neuronal injure. Although many previous researches reported the association among LDL-C, HbA1c and DPN, this research firstly observed the mediating role of HbA1c in the impact of elevated LDL-C levels on the motor fiber conduction velocity of Ulnar (Common peroneal) nerve in individuals with T2DM.

## Conclusion

The effect of elevated LDL-C levels upon the motor fiber of the Ulnar and Common peroneal nerve has been found to be mediated by increased levels of HbA1c in diabetic participants. Moreover, the mediating effect of HbA1c appears to be fully. It has been demonstrated that the effect of elevated LDL-C on the Ulnar nerve and Common peroneal nerve may be contingent upon increased levels of HbA1c in patients with T2DM. In clinical practice, when considering the impact of LDL-C on diabetic peripheral neuropathy, it is insufficient to solely focus on controlling LDL-C. It is imperative to consider plasma glucose control, particularly the level of HbA1c.

## Electronic supplementary material

Supplementary Table1: Pearson correlation between HbA1c and LDL-C in patients with T2DM

## Data Availability

All data in this manuscript have been submitted to The First Affiliated Hospital of USTC for records. Additionally, all ID of recruited patients were also collected for further using. All data are available on reasonable request from corresponding authors.
