# Supplementary material for: The mediating role of HbA1c in the association between elevated low-density lipoprotein cholesterol levels and diabetic peripheral neuropathy in patients with type 2 diabetes mellitus
Source: Lipids Health Dis. 2023 Jul 13;22:102. doi: 10.1186/s12944-023-01865-5 (PMC10339475; doi:10.1186/s12944-023-01865-5)
Supplement: Supplementary file 1 — Supplementary Table1: Pearson correlation between HbA1c and LDL-C in patients with T2DM [file 12944_2023_1865_MOESM1_ESM.docx]

Supplementary Table1: Pearson correlation between HbA1c and LDL-C in patients with T2DM

|  | R | *P* |
| --- | --- | --- |
| In all patients | 0.210 | 0.022^*^ |
| In patients with DPN | 0.283 | 0.022^*^ |

^*^*P* < 0.05

Abbreviations: HbA1c, glycosylated hemoglobin; LDL-C, low density lipoprotein cholesterol; T2DM, type 2 diabetes mellitus.
